# Prospective cohort study of radiotherapy with concomitant and adjuvant temozolomide chemotherapy for glioblastoma patients with no or minimal residual enhancing tumor load after surgery

**DOI:** 10.1007/s11060-012-0798-3

**Published:** 2012-02-04

**Authors:** Walter Stummer, Thomas Meinel, Christian Ewelt, Peter Martus, Olga Jakobs, Jörg Felsberg, Guido Reifenberger

**Affiliations:** 1 Department of Neurosurgery, University of Münster, Albert-Schweitzer Campus 1, Geb. 1 A, 48149 Münster, Germany; 2 Clinstud GmbH, Steidle Str. 10a, 86551 Aichach, Germany; 3 Charité Campus Benjamin Franklin and Campus Mitte Berlin, Institut für Biometrie und Klinische Epidemiologie, Charitéplatz 1, 10098 Berlin, Germany; 4 Department of Neuropathology, Heinrich Heine University, Moorenstr. 5, 40225 Düsseldorf, Germany

**Keywords:** Glioblastoma, O6-methylguanine-DNA methyltransferase, Radiochemotherapy, Resection, Survival, Temozolomide

## Abstract

Survival of glioblastoma patients has been linked to the completeness of surgical resection. Available data, however, were generated with adjuvant radiotherapy. Data confirming that extensive cytoreduction remains beneficial to patients treated with the current standard, concomitant temozolomide radiochemotherapy, are limited. We therefore analyzed the efficacy of radiochemotherapy for patients with little or no residual tumor after surgery. In this prospective, non-interventional multicenter cohort study, entry criteria were histological diagnosis of glioblastoma, small enhancing or no residual tumor on post-operative MRI, and intended temozolomide radiochemotherapy. The primary study objective was progression-free survival; secondary study objectives were survival and toxicity. Furthermore, the prognostic value of O6-methylguanine-DNA methyltransferase (*MGMT*) promoter methylation was investigated in a subgroup of patients. One-hundred and eighty patients were enrolled. Fourteen were excluded by patient request or failure to initiate radiochemotherapy. Twenty-three patients had non-evaluable post-operative imaging. Thus, 143 patients qualified for analysis, with 107 patients having residual tumor diameters ≤1.5 cm. Median follow-up was 24.0 months. Median survival or patients without residual enhancing tumor exceeded the follow-up period. Median survival was 16.9 months for 32 patients with residual tumor diameters >0 to ≤1.5 cm (95% CI: 13.3–20.5, *p* = 0.039), and 13.9 months (10.3–17.5, overall *p* < 0.001) for 36 patients with residual tumor diameters >1.5 cm. Patient age at diagnosis and extent of resection were independently associated with survival. Patients with *MGMT* promoter methylated tumors and complete resection made the best prognosis. Completeness of resection acts synergistically with concomitant and adjuvant radiochemotherapy, especially in patients with *MGMT* promoter methylation.

**Electronic supplementary material:**

The online version of this article (doi:10.1007/s11060-012-0798-3) contains supplementary material, which is available to authorized users.

## Introduction

Cytoreductive surgery for glioblastoma is generally assumed to be beneficial. However, data supporting this assumption are based on studies evaluating the extent of surgical resection in glioblastoma patients treated by adjuvant radiotherapy [1–3]. In 2005, radiotherapy with concomitant and adjuvant temozolomide was established as the standard of care in glioblastoma after the EORTC 26981–22981/NCIC CE.3 trial [4, 5]. In this trial patients with “complete” resections seemed to benefit more than those with “incomplete” resections [5]. However, radicality was not assessed on the basis of imaging, but rather on the judgement of the surgeon, which is known to overestimate the extent of resection [6]. Therefore, the concept that extensive cytoreductive surgery in glioblastoma is still necessary requires verification in a study with post-operative imaging, because the benefits of adding concomitant and adjuvant temozolomide to radiotherapy might overcome benefits derived from extensive resection, with its inherent risks. On the other hand, it may also be that cytoreduction of glioblastoma and removal of residual tumor tissue might enhance the efficacy of radiochemotherapy, thus acting synergistically [7].

We therefore performed a non-interventional cohort study addressing this question, because a randomized study with different degrees of resection would be unfeasible. To minimize the effect of patient-dependent factors on the extent of resection, we required investigators to include patients with small residual contrast-enhancing tumor remnants or no residual tumor. It has been demonstrated [1, 6], that a number of patient-dependent factors may affect the extent of resection, foremost age and Karnofsky performance score (KPS). These factors, which affect resection may also affect survival, and may confound interpretation of data concerning the effect of resection on outcome for retrospective patient cohorts.

Additionally, we assessed O6-methylguanine-DNA methyltransferase (*MGMT*) promoter methylation status [8] in a subset of patients to determine its interaction with resection and outcome.

## Patients and methods

Nineteen centers participated in this prospective, non-interventional cohort study. The study included glioblastoma patients older than 18 years, without previous tumor-specific therapy and without factors precluding radiochemotherapy (hemoglobin ≥ 10 g/dl, neutrophil granulocytes ≥ 1.5 × 109/l, thrombocytes ≥ 100 × 109/l). Early post-operative MRI within 72 h after surgery was required to be indicative of either no residual contrast-enhancing tumor or only minimal residual tumor not exceeding 1.5 cm in diameter (RECIST, http://www.recist.com). RECIST assessments are easier to perform than volumetric methods and have been reported to correlate with overall survival similar to volumetric methods [9–11]).

To assess protocol adherence, MRIs were reviewed in a blinded fashion by one of the authors (T.M.). If tumor diameters were larger than 1.5 cm, patients were analyzed separately. The safety analysis included all patients documented in the study that had begun concomitant radiochemotherapy, irrespective of whether an early post-operative MRI was available.

Participating surgeons were asked to submit paraffin-embedded tumor tissue samples for methylation-specific PCR analysis of the MGMT promoter methylation [12] and central histopathology review at the Brain Tumor Reference Center of the German Society of Neuropathology and Neuroanatomy in Düsseldorf (G.R., J.F.). However, because this was a non-interventional cohort study, submission of tissue samples for reference pathology was not mandatory and samples could not be collected from all patients.

Primary study objectives were to determine progression-free survival overall and stratified by residual tumor volumes based on MRI compared with a well-defined historical cohort of patients with surgery and radiotherapy [1]. Secondary study objectives were to determine overall survival (last assessment 12 months after recruitment of last patient) and toxicity according to CTC criteria.

Therapy data were collected until radiographic tumor progression or until six cycles of adjuvant temozolomide were completed. Follow-up MRIs were performed at three-monthly intervals or at clinical deterioration until radiological progression. Progression was defined as an increase of enhancing tumor diameter by 25% or the appearance of new contrast-enhancing lesions. Survival data, defined as the time between resection and death, were collected for all patients. Patients alive at the time of final analysis were censored at the date of last patient contact.

Patients with available *MGMT* promoter methylation data and early post-operative MRI were stratified according to the extent of resection and *MGMT* status for assessing the combined effect of both of these on survival.

Concomitant radiochemotherapy followed by temozolomide chemotherapy was performed according to the current standard of care. During the concomitant phase patients were treated by radiotherapy (58–60 Gy, daily fractions: 2 Gy) with temozolomide at a dose of 75 mg/m^2^/day. Approximately four weeks after completion of radiotherapy, monotherapy with temozolomide was initiated, beginning with 150 mg/m^2^ per day for five of 28 days during the first cycle, thereafter, if tolerated, with 200 mg/m^2^ during five of 28 days for a total of six cycles.

## Biometry and statistical methods

The sample size was determined to detect a 30% increase of PFS of the entire group of patients compared with the known PFS of approximately 6 months [1] for the cohort operated on with 5-ALA in the ALA study [1] (with 65% complete resections and adjuvant radiotherapy), under the assumption that resection success would be similar in our cohorts, i.e. to detect a PFS of 7.8 months. The type 1 error was 0.05 (two-sided), the type 2 error 0.20, recruitment time 2 years, minimum follow up 1 year, and assumed drop out 25%. Thus, 180 patients had to be recruited to obtain 135 evaluable patients. Sample size was estimated by use of a self-written program implementing the Schoenfeld–Richter nomogram [13].

Univariate statistical analysis was performed using Kaplan–Meier estimates and log rank tests. Multivariable Cox proportional hazards models were used where applicable. Statistical evaluation was performed using SPSS software version 15.

Data collection was performed by Clinstud CRO, Wedel, Germany, and data analysis by the Department of Biometry and Clinical Epidemiology, Charité Berlin (P.M., O.J.). Patients gave informed consent, as required by local ethics review committees in this non-interventional study and all data were entered into anonymous case report forms by local co-investigators before being collected by the study office. Because this was a non-interventional cohort study, no source data verification was performed by monitors.

## Patient cohorts

Patients were recruited between May 2006 and October 2007. Of 180 patients initially reported to the study office, fourteen patients who did not go on to have radiochemotherapy were excluded from the analysis (one patient refused to participate further, for eight patients progression occurred before the start of adjuvant therapy, four patients refused adjuvant therapy, and one patient suffered complicating sigmadiverticulitis precluding radiochemotherapy).

Early post-operative imaging was performed for the remaining 166 patients. For 23 patients, however, the MR images were not evaluable for residual contrast-enhancing tumor by the reference radiologist (missing t1 without contrast, *n* = 6; missing t1 with contrast, *n* = 6; unenhanced CT only, *n* = 3; no MRI available, *n* = 2; cavity obscured by hemorrhage, *n* = 1; pre-OP missing with ambiguous post-OP MRI because of hemorrhagic changes, *n* = 1; bihemispheric lesions, *n* = 1, images not obtainable for reference assessment, *n* = 3). These patients were included in the safety analysis set only. Thus, a total of 143 patients had evaluable early post-operative MRI and concomitant therapy. Of these, 75 patients had no residual contrast-enhancing tumor, 32 patients had residual tumor with a diameter of >0 to 1.5 cm, and 36 patients had a residual tumor load >1.5 cm in diameter. *MGMT* promoter methylation status could be determined in 79 of the 143 patients (55%) with evaluable early post-operative MRI.

Mean tumor diameter for patients with residual tumor was 1.07 ± 0.35 cm (SD, range 0.3–1.5; median 1.1 cm). For patients with tumors >1.5 cm, the mean diameter of residual tumor tissue was 2.7 cm (±1.1 cm, median 2.4 cm, range 1.6–7.1 cm).

## Results

### Safety

The characteristics of patients entered into the safety analysis, including toxic adverse events, are provided in Supplementary Table 1.

### Overall outcome

Median progression-free survival for all 143 patients with evaluable post-operative MRI was 10.4 months (95% CI: 8.1–12.8 months). Thus the primary analysis according to the statistical design—prolongation of PFS longer than 6 months as determined by the lower confidence limit—was successful. Overall survival was 19.4 months (95% CI: 15.6–23.3 months). If those patients with non-evaluable or missing early post-operative MRI were included for sensitivity purposes, median overall survival was comparable (19.0 months, 15.9–22.1 months), thus making systematic distortion of outcome data by omission of these patients unlikely. Median follow up for survival was 24.0 months.

## Outcome stratified by resection status

### Effect of pre-operative factors on extent of resection

To assess whether patient-dependent factors affected extent of resection, which would confound interpretation of outcome data, pre-operative patient (age, KPS) and tumor characteristics (e.g. size, location, extent of edema) that might have affected the decision or ability to perform more or less extensive resections were analyzed. Location was substratified by the factors hemisphere, frontal, temporal, parietal, occipital location, whether more than one brain lobe was involved, whether contrast-enhancing tumor reached the ventricle, and by eloquence. The last was the assessment of the surgeon based on proximity or infiltration of language, motor or visual cortex, or associated tracts. In multivariate analysis (Table 1), only the extent of associated t2 signal abnormality (“edema”, defined as none, ≤2 or >2 cm) was a significant predictive factor for the extent of resection (OR 0.376, 95% CI: 0.153–0.924, *p* = 0.033).

**Table 1:** Factors predicting the extent of resection of enhancing tumor (*n* = 143)

Factor	Univariate			Multivariate		
	OR	95% CI	*p*	OR	95% CI	*p*
Right hemisphere^a^	1.705	0.874–3.328	0.118	0.800	0.031–20.78	0.89
Left hemisphere^a^	0.581	0.297–1.138	0.113	0.316	0.012–8.243	0.48
Frontal location^a^	1.042	0.492–2.206	0.914	0.234	0.039–1.409	0.11
Occipital location^a^	1.107	0.460–2.664	0.820	0.877	0.210–3.656	0.86
Parietal location^a^	1.207	0.562–2.591	0.629	0.857	0.226–3.252	0.820
Temporal location^a^	0.667	0.344–1.295	0.232	0.250	0.056–1.124	0.071
Tumor restricted to single lobe^a^	1.977	0.453–8.623	0.364	3932	0.617–25.038	0.15
Midline shift^a^	0.555	0.281–1.094	0.089	0.452	0.130–1.567	0.21
In eloquent region^a, c^	1.092	0.559–2.134	0.796	1313	0.479–3.598	0.6
Contrast enhancement reaches ventricle^a^	0.396	0.198–0.794	0.009	0.403	0.141–1.158	0.091
Extent of cerebral edema^b^	0.559	0.309–1.010	0.054	0.376	0.153–0.924	0.033
Tumor size^d^	1.368	1.071–1.746	0.012	1172	0.763–1.798	0.47
Age^d^	0.997	0.968–1.027	0.589	0.989	0.947–1.033	0.62
Gender^a^	1.025	0.519–2.022	0.944	1468	0.542–3.972	0.45
Pre-OP KPS^a^	0.940	0.487–1.814	0.854	0.804	0.328–1.973	0.63

^a^Category

^b^None, ≤2 cm, >2 cm

^c^Motor, language, visual (as assessed by surgeon)

^d^Continuous

### Adjuvant therapy

The median time from the date of resection to initiation of radiochemotherapy was 28 days (mean: 30 days) and the median duration of radiotherapy was 44 days (mean: 44.4 days; Supplementary Table 1), which was similar to the duration of concomitant chemotherapy with temozolomide (median: 44 days, mean: 43.9 days). For 77 patients the initial dose of 150 mg/m^2^ was increased to 200 mg/m^2^.

Stratification of patients by residual tumor load revealed no differences concerning radiotherapy and concomitant chemotherapy, either regarding timing or dose. However, in the adjuvant phase of chemotherapy, patients with complete resections received more cycles of temozolomide (*p* = 0.004; Supplementary Table 3) with 38.6% of patients with residual tumor completing six cycles in comparison with 60.3% of patients without residual tumor.

### Progression-free survival and overall survival

In univariate analysis, residual tumor on post-operative MRI, KPS, and pre-operative tumor size were predictors of survival. In multivariate analysis, only age and residual tumor were significant (Table 2). Extent of associated t2 signal abnormality (“edema”), the only factor independently associated with extent of resection, was not predictive of survival.

**Table 2:** Univariate and multivariate analysis of factors predicting survival (*n* = 143)

Factor	Univariate HR	Univariate p	Multivariate HR	Multivariate p
Residual tumor^a^ (0; ≤1.5 cm; >1.5 cm)	2.285	0.000	3.097	0.000
Age^b^	1.014	0.126	1.027	0.045
KPS^a^	1.576	0.022	1.495	0.105
Pre-OP tumor size^b^	1.196	0.008	1.125	0.226
Involvement of ≥2 lobes^a^	1.227	0.359	1.168	0.563
Right hemisphere^a^	0.969	0.878	–	–
Left hemisphere^a^	1.164	0.465	–	–
Frontal location^a^	1.246	0.341	–	–
Occipital location^a^	0.661	0.217	–	–
Parietal location^a^	0.943	0.799	–	–
Temporal location^a^	1.177	0.429	–	–
Eloquent location^a, c^	0.972	0.892	0.921	0.767
Cerebral edema^b, d^	1.161	0.438	1.233	0.422

^a^Category

^b^Continuous

^c^Motor, language, visual (as assessed by surgeon)

^d^None, ≤2 cm, >2 cm

Progression-free and overall survival were investigated separately in the 107 per-protocol patients with less than 1.5 cm of residual tumor on post-operative MRI, stratified by 0 (*n* = 75) vs. >0 to ≤1.5 cm (*n* = 32) residual tumor diameters.

**Fig. 1:**
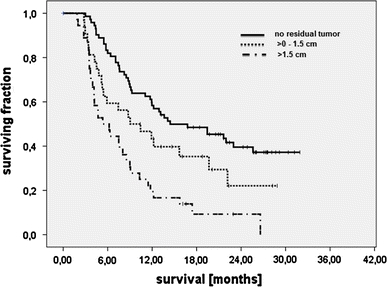
Progression-free survival (*n* = 143), stratified by enhancing residual glioblastoma loads determined in accordance with RECIST criteria (*p* < 0.001)

Progression-free survival was 13.5 months (9.9–17.1) for patients with residual tumor of up to 1.5 cm in diameter and 18.7 months (16.3–21.3, *p* = 0.058; Fig. 1) for patients without residual contrast-enhancing tumor. With residual tumor diameters of >1.5 cm, progression-free survival was shorter (8.5 months, 6.2–10.9, overall *p* < 0.001, Fig. 1).

**Fig. 2:**
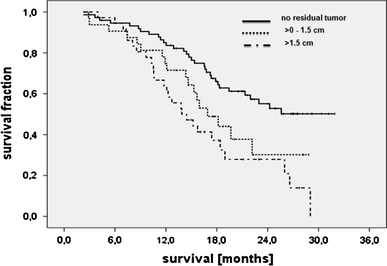
Overall survival (*n* = 143), stratified by enhancing residual glioblastoma loads determined in accordance with RECIST criteria (*p* < 0.001)

Median follow-up duration for survival was 24.0 months. Median overall survival surpassed the follow-up period for patients without residual enhancing tumor, with a mean survival at the time of final assessment of 23.6 months (range: 21.4–25.8 months). Median survival was 16.9 months (range 13.3–20.5 months, *p* = 0.039) for patients with residual contrast-enhancing tumor of >0 to ≤1.5 cm diameter. In comparison patients with >1.5 cm residual tumor diameter did worse (13.9 months; range: 10.3–17.5, overall *p* < 0.001, Fig. 2).

### *MGMT* promoter methylation

**Fig. 3:**
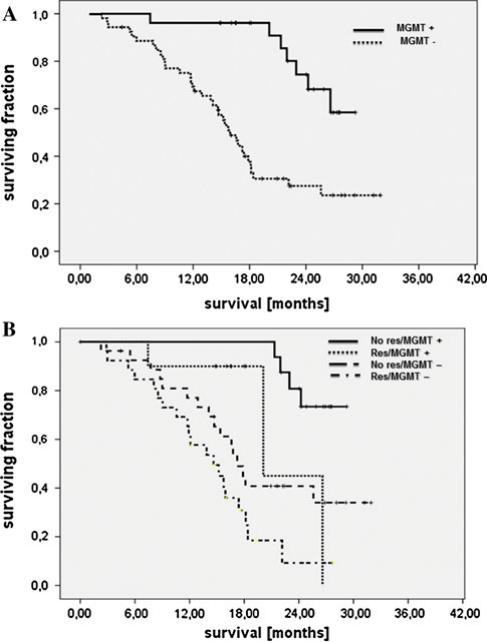
**a** Patients stratified by MGMT status (*MGMT +*, methylated MGMT promoter; *MGMT −*, unmethylated MGMT promoter; *p* = 0.001) (*n* = 79), **b** Patients stratified by resection and MGMT promoter methylation (*No res*, no residual tumor; *Res*, residual tumor; *MGMT +*, methylated MGMT promoter; *MGMT −*, unmethylated MGMT promoter; overall *p* = 0.0005) (*n* = 79)

*MGMT* promoter methylation status was determined for 79 of 143 patients. Methylated promoter was found in 29 patients (36.7%) and unmethylated promoter in 50 patients (63.3%). Median overall survival of patients with unmethylated tumors was 16.6 months (95% CI: 13.8–19. 3). Median survival was not reached for patients with methylated tumors (mean survival estimated at the end of the observation period: 26.4 months; 24.5–28.3, *p* = 0.0005, Fig. 3a). Patients with methylated tumors and complete resection tended to have the best survival (median not reached, mean: 27.6 months, 95% CI: 26.3–29.0 months), whereas patients with MGMT unmethylated tumors and incomplete resection did worst (median 13.9 months, 10.2–17.5 months, *p* = 0.000), as is apparent from Fig. 3b. Both methylation status and extent of resection were independently related to survival (MGMT promoter methylation: *p* = 0.000, HR = 4.40, 95% CI: 1.93–10.1; extent of resection: *p* = 0.009, HR = 2.37, 1.24–4.50).

## Discussion

This was a prospective cohort study designed to test the effect of extensive cytoreductive surgery for glioblastoma when combined with temozolomide radiochemotherapy followed by adjuvant temozolomide. The question is important, because extensive cytoreductive surgery for glioblastoma would entail unnecessary risks if concomitant radiochemotherapy were effective for small volumes of residual tumor. On the other hand, it may well be that radiochemotherapy followed by adjuvant temozolomide is more effective when all contrast-enhancing tumor is removed surgically. One explanation may be that enhancing regions of malignant glioma on MRI are hypoxic. Hypoxia is associated with aggressive growth and poor response to treatment, including radiotherapy [14, 15]. Hypoxia affects tumor cells by activating genes involved in the adaptation to hypoxic stress, an important aspect of cancer prognosis [16]. Needle electrode studies have shown that oxygenation is lower in glioblastoma than in the surrounding brain tissue [17] and perinecrotic regions in glioblastoma are known to up-regulate hypoxia-inducible factor (HIF) signaling [18] and stain positive for the injectable hypoxia marker EF5 (2-(2-nitro-1*H*-imidazol-1-yl)-*N*-(2,2,3,3,3-pentafluoropropyl)acetamide) [16]. Extensive cytoreduction is likely to remove the central core of resilient, hypoxic, proliferative cells and some of the contrast-enhancing migratory cells in the marginal region, achieving rapid tumor cell removal and thus minimizing the target for adjuvant therapy [19].

We chose a prospective cohort study to address the question of how outcome of radiochemotherapy is related to the extent of resection. Non-randomized cohorts, however, suffer the disadvantage of selection effects for the extent of resection, i.e. differences of resection status dependent on age, tumor location, or KPS, that could confound interpretation. To this end, it has been questioned whether retrospective surgical cohorts were adequately balanced by known prognostic factors such as age and Karnofsky performance score (KPS) [20]. Studies in which the distribution of such factors was assessed revealed that more extensive resection was achieved for younger patients or patients with high KPS scores [1, 6, 21]. Moreover, small and superficially located tumors are more likely to undergo extensive resection, and such tumors may intrinsically have a better prognosis [22, 23]. We therefore conceived our study as suggested by Hess [20], who argued that “In the absence of randomized experiments with well-defined protocols for aggressive and conservative surgery, well-planned and carefully executed prospective observational studies are needed”.

To minimize the effect of patient-dependent factors, for example age, tumor location, tumor size, etc., on the extent of resection, we attempted to limit inclusion to patients with no or small residual tumor loads with a diameter of less than 1.5 cm according to RECIST, based on assessment by surgeons. The reason for using RECIST criteria rather than two-dimensional (McDonalds) or three-dimensional assessments was to simplify, as far as possible, measurement of small residual tumors in this uncontrolled study. Several studies have demonstrated good concordance among RECIST, two-dimensional, and three-dimensional measurements in gliomas [9–11]. Our objective was to include patients with no or minimal residual tumor. In our analysis from the randomized ALA study, median tumor volumes of 1.5 ccm were associated with significantly worse survival [1]. We converted this volume into a diameter (1.43 cm) which we rounded to give 1.5 cm, as used to define upper volume of “small residual tumor” in this study.

Nevertheless, we found central image review to reveal a number of patients with larger residual tumor loads, resulting in our subdivision of patients cohorts with no residual tumor, those with residual tumor less than 1.5 cm in diameter, and those with residual tumor more than >1.5 cm. Analysis of factors that might have resulted in different extent of resection found it to be affected by the extent of cerebral edema, as assessed by t2 signal abnormality. Edema was not a prognostic factor for progression-free or overall survival and thus did not seem to confound interpretation of the effect of resection on survival. Therefore, our strategy of minimizing bias created by different patient-dependent factors, for example age, tumor location, KPS, tumor size, and others, on the extent of surgery seemed effective and enabled more rigorous conclusions regarding the benefit of cytoreductive surgery than possibly afforded by other studies [2, 3, 6, 22, 24–28]. However, the conclusion of a missing effect of these known prognostic factors is only valid for our cohorts, which are subselections of patients. Age and proximity of tumor to eloquent brain, especially, have been identified as independent factors affecting extent of resection in more unselected series [29].

The strategy of including only patients with small residual tumor loads also resulted in adjuvant therapy being very similar when comparing resection groups, with the exception of the number of cycles of adjuvant temozolomide chemotherapy. In our opinion, it is unlikely that this difference would have a detectable effect on survival. Rather, because documentation of therapy was concluded with tumor progression, and patients with residual tumor had early progression, we assume that the differences in the number of cycles are an indicator of prognosis.

The conclusions from our analysis regarding progression-free survival are, however, weakened by the lack of bioptical confirmation, and may be confounded by pseudoprogression, which is observed in 20 to 30% of patients [30]. Because ours was an observational study, therapeutic decisions based on the perception of progression were at the discretion of the individual center therapist. However, pseudoprogression usually occurs during the first three months after radiochemotherapy, and radiological progression in our cohort was observed mostly after this time. Second, the differences between survival were also significant. Also, there was no obligation of participating surgeons to submit tissue for reference histological assessment and central determination of MGMT promoter methylation, again because of the observational nature of our study. This weakens some of our conclusions on the interaction between this molecular predictor and resection status.

Our analysis of overall survival revealed that the cohort of patients without residual enhancing tumor lived longer than patients with enhancing residual tumor, even when comparing no residual with small residual tumor loads (≤1.5 cm diameter). In multivariate analysis, resection was independently predictive of survival. Taken together, our observations suggest a beneficial effect of optimum resection on radiochemotherapy.

Survival in our cohorts was slightly higher than in the resection cohort from the EORTC 26981 study [5] (median: 18.8 months for patients with “complete” resections, and 13.5 months for incomplete resections). In this study, assessment of completeness was not based on imaging but on surgeons’ judgement, which overestimates resection [6]. A more recent phase II study [31] found a PFS of 7.6 months and a OS of 21.1 months in a historical control cohort of glioblastoma patients from UCLA treated by surgery and adjuvant temozolomide radiochemotherapy. However, the latter study did not stratify outcome data by radiological extent of resection, so the effect of resection on the efficacy of radiochemotherapy was not determined. Furthermore, 21% of patients were reported to have had biopsies, 36% subtotal and 43% gross total resections, again making a comparison with our cohort difficult.

Compared with the EORTC 26981 study [4], no particular differences were noted in our cohort regarding radiotherapy (dose, duration) or duration of concomitant therapy and number of cycles of adjuvant radiochemotherapy. In the EORTC 26981 study, duration of concomitant therapy was 42 days (this study 44 days); toxic effects were observed in 5% of patients (this study: 7.2% with grade III or IV leukopenia or thrombopenia), median number of adjuvant cycles was three (this study four cycles), 47% of patients completed six cycles (this study 47.8%), with the exception of dose escalation to 200 mg/m^2^, which was implemented less frequently for patients in our study (54% vs. 67% of patients in the EORTC study).

We found that MGMT promoter methylation and extent of resection were independent predictors of survival. In the subgroup of patients with MGMT promoter methylated tumors *and* complete resections in our study, median survival was not reached during the observation period. Mean survival within the observation period was 27.3 months (95% CI: 25.6–28.9). MGMT promoter methylation also seemed prognostic for patients with incomplete resections, although the number of patients with incomplete resection and promoter methylation was too small to detect statistical significance.

## Conclusions

This observational cohort study supports the importance of cytoreductive therapy for adjuvant temozolomide radiochemotherapy in the treatment of glioblastoma. Thus, surgeons should still attempt to achieve the highest extent of resection, preferably of all contrast-enhancing tumor if safely possible. The prognostic effect of MGMT promoter methylation status seems to be independent of resection, and patients with promoter methylation have the best prognosis, in particular when the entire contrast-enhancing tumor mass is removed.

### Electronic supplementary material

Below is the link to the electronic supplementary material.

Supplementary material 1 (DOCX 20 kb)

Supplementary material 2 (DOC 82 kb)

Supplementary material 3 (DOCX 24 kb)

## Appendix: Participating surgeons

J. Gilsbach, M. Oertel (Neurochirurgische Klinik Med. Einrichtungen Aachen), K. Franz, A. Oszvald (Zentrum für Neurologie und Neurochirurgie Klinikum, Johann Wolfgang Goethe Universität Frankfurt a. M.), T. Stretz (Neurochirurgische Klinik, Zentralklinikum Augsburg), D.K. Böker, U. Nestler (Neurochirurgische Klinik, Medizinisches Zentrum für Neurologie und Neurochirurgie Gießen), F. Oppel, A. Brune (Klinik für Neurochirurgie Evangelisches Krankenhaus, Bielefeld), V. Rhode, A.Giese (Klinik und Poliklinik für Neurochirurgie Georg-August-Universität, Göttingen), J. Schramm, M. Simon (Neurochirurgische Klinik, Universität Bonn), H.J. Meisel, B·C. Kern (Neurochirurgische Abteilung, Berufsgenossenschaftliche Kliniken Bergmannstrost, Halle), A. Unterberg, R. Ahmadi (Neurochirurgische Klinik, Ruprecht-Karls-Universität, Heidelberg), G. Schackert, D. Krex, Klinik für Neurochirurgie, Universitätsklinikum Carl Gustav Carus der TU Dresden), M. Mehdorn, S. Schultka (Campus Kiel, Klinik für Neurochirurgie, Universitätsklinikum Schleswig–Holstein, Kiel), J. Meixensberger, A. Goldammer (Klinik und Poliklinik für Neurochirurgie, Universitätsklinikum, Leipzig), U. Kehler, M. Kämper (Neurochirurgische Abteilung, Asklepios Klinik Altona, Hamburg), C. B. Lumenta, M. Röhrdanz (Abteilung für Neurochirurgie, Krankenhaus Bogenhausen, München), H.H. Steiner, K. Kieselbach (Neurochirurgische Klinik, Klinikum Nürnberg Süd, Nürnberg).
