# Supplementary material for: Prospective cohort study of radiotherapy with concomitant and adjuvant temozolomide chemotherapy for glioblastoma patients with no or minimal residual enhancing tumor load after surgery
Source: J Neurooncol. 2012 Feb 4;108(1):89–97. doi: 10.1007/s11060-012-0798-3 (PMC3337400; doi:10.1007/s11060-012-0798-3)
Supplement: Supplementary file 1 — Supplementary material 1 (DOCX 20 kb) [file 11060_2012_798_MOESM1_ESM.docx]

| **Supplement Table 1:** |  | | |  | | |
| --- | --- | --- | --- | --- | --- | --- |
| Characteristics of patients in the safety analysis set **(N=166)** | | | | | | |
| N=166 | |  | | | |  |
| male | | 101 (61%) | | | |  |
| female | | 65 (39%) | | | |  |
| median age (range) | | 59.5 (21-81) | | | |  |
| **Post-OP KPS** | |  | | | |  |
| KPS 90-100 | | 77 (56%) | | | |  |
| KPS 70-80 | | 55 (40%) | | | |  |
| KPS 50-60 | | 6 (4%) | | | |  |
| Not recorded | | 28 (17%) | | | |  |
| **Location** | |  | | | |  |
| Left-sided tumor | | 74 (46%) | | | |  |
| Right-sided tumor | | 91 (55%) | | | |  |
| Temporal | | 80 (48%) | | | |  |
| Parietal | | 44 (27%) | | | |  |
| Frontal | | 43 (26%) | | | |  |
| Occipital | | 25 (15) | | | |  |
| Involving 2 or more lobes | | 10 (6%) | | | |  |
| pre-OP MRI missing | | 7 (4%) | | | |  |
|  | | | | | |  |
|  |  |  |  |  |  |  |
|  |  |  |  |  |  |  |
| **Supplement Table 1 continued** | | | | | |  |
| **Residual tumor diameter** | | |  | | |  |
| ≤1.5 cm | | | 107 (64%) | | |  |
| >1.5 cm | | | 36 (22%) | | |  |
| No residual tumor | | | 75 (45%) | | |  |
| >0 to ≤1.5cm | | | 32 (19%) | | |  |
| Not evaluable/not available | | | 23 (14%) | | |  |
| **Pre-OP MRI** | | |  | | |  |
| Midline displacement | | | 94 (57%) | | |  |
| Eloquent location | | | 71 (43%) | | |  |
| Language | | | 28 (17%) | | |  |
| Motor | | | 19 (11%) | | |  |
| Optical pathways | | | 18 (11%) | | |  |
| Others | | | 7 (4%) | | |  |
| Extent cerebral edema | | |  | | |  |
| 0 cm | | | 6 (4%) | | |  |
| >0 – 2cm | | | 71 (43%) | | |  |
| >2 cm | | | 84 (51%) | | |  |
| Unknown | | | 5 (2) | | |  |
| Enhancing tumor reaches ventricle | | | 87 (52%) | | |  |
| Tumor diameter | | | 4.7±1.4 | | |  |
| (cm, mean ± SD) | | |  |  |  |  |
| Range (cm) | | | 1.7-8.3 | | |  |
| Pre-OP MRI missing | | | 1 (0.6%) | | |  |
|  | | |  | | |  |
| **Supplement Table 1 continued** | | | | | |  |
| **Adjuvant therapy** | | | | |  |  |
| Time to begin after surgery (d) | | | | | 29.64 ±10.75 |  |
| Median (d) | | | | | 28 |  |
| Duration of radiotherapy (d) | | | | | 44.61±6.84 |  |
| Radiation dose (Gy, avg±SD) | | | | | 59.42±3.26 |  |
| Duration concomitant TMZ chemotherapy (d, avg±SD) | | | | | 44.24±6.97 |  |
| Duration (d, median) | | | | | 44 |  |
| **Interruption of adjuvant chemotherapy n/(N)** | | | | | 9 (166) |  |
| **Week 1** | | | | | 4 (165) |  |
| **Week 2** | | | | | 1 (165) |  |
| **Week 3** | | | | | 1 (164) |  |
| **Week 4** | | | | | 0 (161) |  |
| **Week 5** | | | | | 2 (154) |  |
| **Week 6** | | | | | 1 (75) |  |
| **Adjuvant chemotherapy n(%)** | | | | |  |  |
| 1 cycle [n, (%)] | | | | | 28 (20.2) |  |
| 2 cycles [n, (%)] | | | | | 13 (9.4) |  |
| 3 cycles [n, (%)] | | | | | 13 (9.4) |  |
| 4 cycles [n, (%)] | | | | | 9 (6.5) |  |
| 5 cycles [n, (%)] | | | | | 9 (6.5) |  |
| 6 cycles [n, (%)] | | | | | 66 (47.8) |  |
| N (%) | | | | | 138 (100) |  |
| missing | | | | | 25 |  |
|  | | | | |  |  |
