# Supplementary material for: Prospective cohort study of radiotherapy with concomitant and adjuvant temozolomide chemotherapy for glioblastoma patients with no or minimal residual enhancing tumor load after surgery
Source: J Neurooncol. 2012 Feb 4;108(1):89–97. doi: 10.1007/s11060-012-0798-3 (PMC3337400; doi:10.1007/s11060-012-0798-3)
Supplement: Supplementary file 2 — Supplementary material 2 (DOC 82 kb) [file 11060_2012_798_MOESM2_ESM.doc]

| **Supplement Table 2a:** Summary of toxicities related to radiochemotherapy, stratified by visit (n=166, safety analysis population) | | | | | | | | | | | | |  |  | |
| --- | --- | --- | --- | --- | --- | --- | --- | --- | --- | --- | --- | --- | --- | --- | --- |
|  | | | | | | | | | | | | |  |  | |
| Phase | Visit | | Leukopenia | | | | | Thrombopenia | | | | All toxicities |  | Pregnancy | |
|  |  | | Grade | | | | | Grade | | | |  | SAE |  |  |
|  |  | | I | | II | | III | I | II | III | IV |  |  |  |  |
| intial | Post OP | |  | |  | | 1 |  |  |  |  | 8 | 4 |  | |
| Concomitant therapy | 1 week | |  | |  | |  | 1 |  |  |  | 12 | 6 |  | |
| (weeks after begin of therapy) | 2 weeks | |  | |  | |  |  |  |  |  | 14 |  |  | |
|  | 3 weeks | |  | |  | |  |  | 1 |  |  | 10 | 5 | 1 | |
|  | 4 weeks | |  | |  | |  |  | 1 |  |  | 6 | 1 |  | |
|  | 5 weeks | |  | |  | |  | 1 | 1 |  | 1 | 8 | 5 |  | |
|  | 6 weeks | | 1 | |  | |  | 7 | 3 | 1 |  | 11 | 5 |  | |
|  | 7 weeks | |  | |  | |  | 6 | 2 | 1 | 1 | 6 | 3 |  | |
| Adjuvant phase | 1 month | | 2 | |  | |  | 2 | 2 | 1 |  | 22 | 6 |  | |
| (months after begin of phase) | 2 months | | 2 | |  | |  | 3 | 1 | 3 | 1 | 15 | 4 |  | |
|  | 3 months | | 3 | | 1 | |  | 1 | 1 | 1 |  | 17 | 5 |  | |
|  | 4 months | | 2 | | 1 | |  | 3 | 2 | 1 |  | 10 | 2 |  | |
|  | 5 months | | 2 | | 1 | |  | 1 |  |  |  | 8 | 4 |  | |
|  | 6 months | | 5 | |  | |  | 2 | 1 |  |  | 3 | 1 |  | |
| 12 months post surgery |  | |  | |  | |  |  |  |  |  | 3 | 2 |  | |
| end |  | |  | | 1 | |  |  | 1 |  |  | 13 | 18 |  | |
|  |  | |  | |  | |  |  |  |  |  |  |  |  | |
|  | |  | | | |  | | | | | | | | |  |
| **Supplement Table 2b:** Severe adverse events as reported by study physicians (only SAE frequency >1), safety analysis set | | | | | | | | | | | | | | |  |
| N=166 | | | | n | | | |  | | | | | | |  |
| Deterioration of general condition | | | | 7 | | | |  | | | | | | |  |
| Hospitalisation | | | | 9 | | | |  | | | | | | |  |
| Focal seizure | | | | 5 | | | |  | | | | | | |  |
| Impaired wound healing | | | | 4 | | | |  | | | | | | |  |
| Generalized seizure | | | | 4 | | | |  | | | | | | |  |
| Pulmonary embolism | | | | 4 | | | |  | | | | | | |  |
| Pancytopenia | | | | 3 | | | |  | | | | | | |  |
| Hospitalisation due to progression | | | | 3 | | | |  | | | | | | |  |
| Death | | | | 2 | | | |  | | | | | | |  |
| Thrombopenia | | | | 2 | | | |  | | | | | | |  |
| Hydrocephalus | | | | 2 | | | |  | | | | | | |  |
| Increase of cerebral edema | | | | 2 | | | |  | | | | | | |  |
|  | | | |  | | | |  | | | | | | |  |
|  | | | |  | | | |  | | | | | | |  |
|  | | | |  | | | |  | | | | | | |  |
| **Supplement Table 2c:** Adverse events as reported by study surgeons, safety analysis set (AE with frequency >2 only) | | | | | | | | | | | | | | |  |
| Fatigue | | 18 | | | |  | | | | | | | | |  |
| Headache | | 13 | | | |  | | | | | | | | |  |
| Obstipation | | 10 | | | |  | | | | | | | | |  |
| Leucocytosis | | 9 | | | |  | | | | | | | | |  |
| Nausea | | 8 | | | |  | | | | | | | | |  |
| Appetite diminished | | 7 | | | |  | | | | | | | | |  |
| Granulocytosis (neutrophils) | | 5 | | | |  | | | | | | | | |  |
| Lymphopenia | | 4 | | | |  | | | | | | | | |  |
| Insominia | | 4 | | | |  | | | | | | | | |  |
| Low hemoglobine | | 4 | | | |  | | | | | | | | |  |
| Alopecia | | 3 | | | |  | | | | | | | | |  |
| Depression | | 3 | | | |  | | | | | | | | |  |
| Vertigo | | 3 | | | |  | | | | | | | | |  |
| Thrombopenia | | 3 | | | |  | | | | | | | | |  |
| Pruritus | | 3 | | | |  | | | | | | | | |  |
| Taste changes | | 3 | | | |  | | | | | | | | |  |
| Epileptic seizure | | 3 | | | |  | | | | | | | | |  |
| Gamma GT increased | | 3 | | | |  | | | | | | | | |  |
| Low hematocrit | | 3 | | | |  | | | | | | | | |  |
|  | |  | | | |  | | | | | | | | |  |
