# Supplementary material for: Prospective cohort study of radiotherapy with concomitant and adjuvant temozolomide chemotherapy for glioblastoma patients with no or minimal residual enhancing tumor load after surgery
Source: J Neurooncol. 2012 Feb 4;108(1):89–97. doi: 10.1007/s11060-012-0798-3 (PMC3337400; doi:10.1007/s11060-012-0798-3)
Supplement: Supplementary file 3 — Supplementary material 3 (DOCX 24 kb) [file 11060_2012_798_MOESM3_ESM.docx]

| **Supplement Table 3** Adjuvant therapy stratified by degree of resection (n=143) | | | | | | |
| --- | --- | --- | --- | --- | --- | --- |
| **charateristic** | | **All patients**  **(n=143)** | | **0 cm residual tumor [n (%)]** | **>0 cm residual tumor [n (%)]** | **p** |
| Radiotherapy | |  | |  |  |  |
| Time to begin (d) | |  | |  |  | 0.83 |
| Average+-SD | | 30.1±10.7 | | 29.5±9.79 | 30.7±11.7 |  |
| Median | | 28 | | 28 | 28 |  |
| Minimum | | 9 | | 13 | 9 |  |
| Maximium | | 69 | | 60 | 69 |  |
| Missing | | 0 | | 0 | 0 |  |
| N | | 143 | | 75 | 68 |  |
|  | |  | |  |  |  |
| Duration (d) | |  | |  |  | 0.45 |
| Average+-SD | | 44.4±6.4 | | 44.5±7.83 | 44.3±4.53 |  |
| Median | | 44 | | 44 | 44,5 |  |
| Minimum | | 25 | | 25 | 26 |  |
| Maximium | | 93 | | 93 | 54 |  |
| Missing | | 5 | | 5 | 0 |  |
| N | | 138 | | 70 | 68 |  |
|  | |  | |  |  |  |
|  | |  | |  |  |  |
|  | |  | |  |  |  |
|  | |  | |  |  |  |
|  | |  | |  |  |  |
|  | |  | |  |  |  |
| **Supplement Table 3 continued** | | | |  |  |  |
| Total dose (Gy) | |  | |  |  |  |
| Average+-SD | | 59.4±3.4 Gy | | 59.6±0.95 | 59.2±4.90 | 0.87 |
| Median | | 59.4 | | 59.4 | 59.4 |  |
| Minimum | | 32.4 | | 54 | 32.4 |  |
| Maximium | | 70 | | 60 | 70 |  |
| Missing | | 66 | | 35 | 31 |  |
| N | | 77 | | 40 | 37 |  |
|  | |  | |  |  |  |
| Chemotherapy | |  | |  |  |  |
| Duration of concomitant therapy | |  | |  |  |  |
| Average+-SD | | 43.9±6.3 | | 44.2±7.43 | 43.6±5.04 | 0.57 |
| Median | | 44 | | 43 | 44 |  |
| Minimum | | 27 | | 27 | 26 |  |
| Maximium | | 94 | | 94 | 54 |  |
| Missing | | 6 | | 6 | 0 |  |
| N | | 137 | | 69 | 68 |  |
|  | |  | |  |  |  |
|  | |  | |  |  |  |
|  | |  | |  |  |  |
|  | |  | |  |  |  |
|  | |  | |  |  |  |
| **Supplement Table 3 continued** | | | |  |  |  |
| Interruption adjuvant chemotherapy n (N) | | | | | | |
| Week 1 | 3/143 | | 3 of 74 | | 0 of 68 | 0.25 (fisher’s exact) |
| Week 2 | 0 (143) | | 0 of 74 | | 0 of 68 | - |
| Week 3 | 1 (143) | | 1 of 74 | | 0 of 68 | 1.000 |
| Week 4 | 0 (138) | | 0 of 70 | | 0 of 68 | 1.000 |
| Week 5 | 1 (136) | | 0 of 69 | | 1 of 67 | 0,5 |
| Week 6 | 0 (67) | | 0 of 33 | | 0 of 34 | - |
|  |  | |  | |  |  |
| Number of cycles of adjuvant chemotherapy prior to progression | | | | | |  |
| 1 cycle | 25 (20.8%) | | 8 (12.7) | | 17 (29.8) | 0.004 (Chi Square test) |
| 2 cycles | 13 (10.8%) | | 4 (6.3) | | 9 (15.8) |  |
| 3 cycles | 10 (8.3%) | | 3 (4.8) | | 7 (12.3) |  |
| 4 cycles | 6 (5%) | | 6 (9.5) | | 0 (0) |  |
| 5 cycles | 6 (5%) | | 4 (6.3) | | 2 (3.5) |  |
| 6 cycles | 60 (50%) | | 38 (60.3) | | 22 (38.6) |  |
| N (%) | 120 (100) | | 63 (100) | | 57 (100) |  |
| Missing | 23 | | 12 | | 11 |  |
|  |  | |  | |  |  |
|  |  | |  | |  |  |
|  |  | |  | |  |  |
